# The Swiss Ecotox Centre: bridging the gap between research and application

**DOI:** 10.1186/s12302-018-0147-z

**Published:** 2018-05-16

**Authors:** Inge Werner

**Affiliations:** Swiss Centre for Applied Ecotoxicology, Überlandstrasse 133, 8600 Dübendorf, Switzerland

**Keywords:** Ecotoxicology, Environmental risk, Chemicals, Societal needs, Science-policy interface

## Abstract

The Swiss Centre for Applied Ecotoxicology (Ecotox Centre) was created in recognition of the urgent societal need to provide expertise, education and tools for assessing the risks and effects of anthropogenic chemicals in the environment. Founded in 2008, the Ecotox Centre conducts applied, practice-oriented research in the areas of aquatic (water and sediment) and terrestrial (with focus on soil) ecotoxicology, and provides further education and consulting services to its stakeholders. To date, its most important activities focus on (1) the validation and standardization of bioassays for use in monitoring of water, sediment or soil quality and (2) the development of tools for retrospective risk assessment, including approaches to assess mixture risk.

## Introduction

As more and more anthropogenic chemicals are released into the environment threatening the functioning of ecosystems worldwide, the importance of the scientific field of ecotoxicology is growing. Such expertise is needed by authorities in charge of assessing chemical risk and safeguarding the environment, as well as by private enterprise facing regulation of hazardous chemicals.

In recognition of the urgent need for expertise in applied ecotoxicology, the Ecotox Centre was founded in 2008 by request of the Federal Council and Parliament of Switzerland as the Swiss Centre for Applied Ecotoxicology^1^ with a centre-specific mandate. The Centre is headquartered at Eawag in Dübendorf with a subsidiary at École polytechnique fédérale de Lausanne (EPFL). Basis and point of reference for the development and structure of the Ecotox Centre is the “Report of the Federal Council on independent toxicology research in Switzerland”^2^ of 2007. It defines the fundamental role and responsibilities of the Ecotox Centre, which are:

- Providing practical education and training in the field of ecotoxicology.
- Development of ecotoxicological testing and assessment methods.
- Implementation of screening programs for early detection of environmental risks, and development of the methodology required for this purpose.
- Consulting, function as a hub for questions in the field of ecotoxicology.
- Evaluation of chemicals.
- Participation in national and international expert groups.
- Communication of ecotoxicological concerns.

**Fig. 1:**
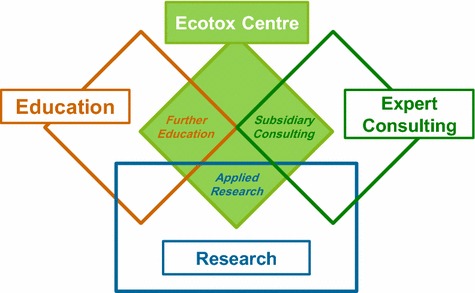
Positioning of the Ecotox Centre with respect to its principal tasks

Now in its 10th year of existence, the Ecotox Centre has successfully established itself in Switzerland, is internationally recognized, and fulfils the mandate of the Federal Council. In close collaboration with authorities in Switzerland and abroad, as well as national and international research groups, the Centre conducts applied research in the areas of aquatic (water and sediment) and terrestrial (with focus on soil) ecotoxicology, and provides further education and consulting services to its stakeholders. It has become a hub for research and development, service, information and education in applied, practice-oriented ecotoxicology (Fig. 1).

## Organizational Structure of the Centre

**Fig. 2:**
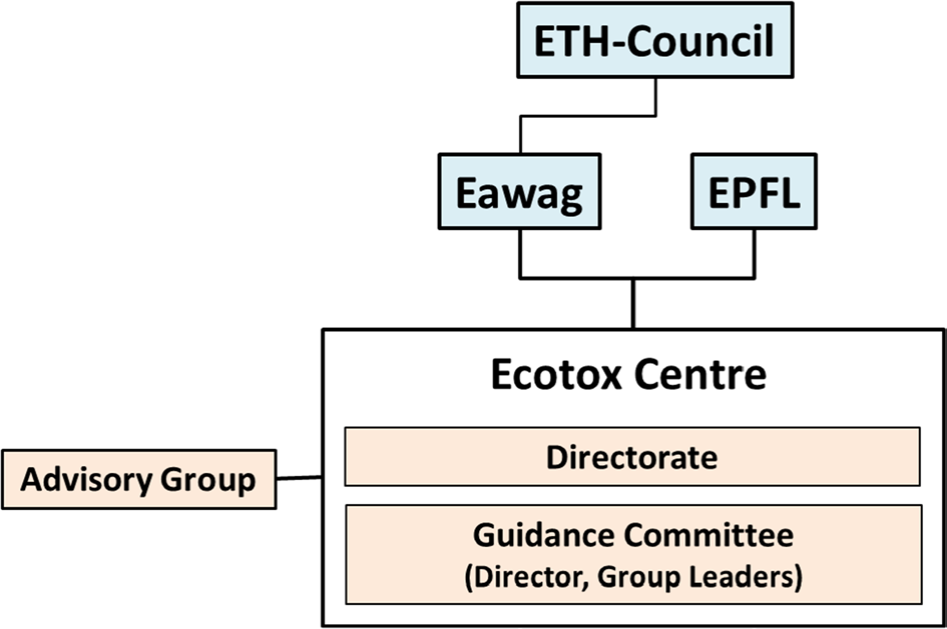
Organizational structure of the Ecotox Centre within the ETH domain

The Ecotox Centre is an independent institution embedded in the ETH (Eidgenössische Technische Hochschule) domain, and associated with Eawag, the Swiss water research institute and EPFL. It is administratively tied and annually reports to Eawag (Fig. 2). Eawag in turn reports on behalf of the Ecotox Centre to the ETH Council. The Centre’s Directorate consists of one representative each from Eawag and EPFL, and the Centre Director. An external advisory group provides stakeholder input, reviews and comments on strategic plans, and is responsible for the evaluation of the Centre at regular (4 year) intervals.

The Centre currently receives approximately 2.3 Mio CHF in Federal funds annually. In addition it receives extramural funds from a variety of sources. It currently employs approximately 20 permanent and temporary staff. Four different areas of expertise exist at the Ecotox Centre at present: (1) Soil ecotoxicology; (2) Sediment ecotoxicology; (3) Aquatic ecotoxicology and (4) Risk assessment. A chemist provides analytical support.

In addition to office and lab space, Eawag provides administrative services in accounting and personnel, IT and technical support, facilities for Ecotox courses, mail services, and support from the communication department. Some laboratory equipment is shared with Eawag research departments. Besides space, EPFL provides the infrastructure available at the Environmental Engineering Institute—IIE, where the Ecotox Centre is located, as well as support from IT, technical services and the communications department. Both institutions receive overhead funds from the Ecotox Centre.

## Activities

Centre staff currently devotes about 50–60% of work time to activities on ecotoxicological effect assessment (e.g. projects involving bioassays, participation in standardization efforts), and 20% to projects on environmental hazard and risk assessment (e.g. derivation of environmental quality standards, mixture assessment). Approximately 10–20% of total work time is spent on activities in further education, training, outreach and information, and 10% on Centre administration.

Projects carried out at the Ecotox Centre must have an applied focus. The Centre has the ability to carry out projects on its own initiative where a need has been identified. Projects generally focus on topics related to ecotoxicological hazard and risk assessment, the application of bioassays in environmental monitoring and the development and optimization of evaluation methods. The majority are carried out in collaboration with external partners from government agencies, practitioners from cantonal agencies, academic research groups and private companies.

### Quantifying toxicity—bioassays for routine environmental monitoring and effect assessment

The validation and standardization of bioassays for use in the monitoring of water, sediment or soil quality is one of the most important areas of applied ecotoxicological research. Bioassays are essential for the integrative quantification of toxic effects due to chemical mixtures, which are the norm in the environment.

#### Monitoring the efficiency of wastewater treatment technologies

The Ecotox Centre played an important role in the ecotoxicological evaluation of wastewater effluents and advanced treatment technologies to reduce water pollution due to organic micropollutants. In projects such as “Strategy Micropoll”, selected methods were used for monitoring effects in treated wastewater [28]. The Centre thus contributed to the scientific evidence, based on which new regulation was approved in 2013 by the Swiss legislature^3^ requiring major wastewater treatment plants (or those discharging into sensitive habitat) to upgrade to advanced treatment using ozonation or activated carbon. Guidance for using bioassays to monitor wastewater effluent impacted streams for estrogenic and herbicidal compounds is provided by Kienle et al. [24, 25].

Other studies focused on comparing treatment methods for wastewater using bioassay data. In the EU project “Demonstration of promising technologies to address emerging pollutants in water and wastewater (DEMEAU)”, carefully selected bioassays were applied for the evaluation of technologies to eliminate and monitor aquatic micropollutants (e.g. [35]. The study “Biological post-treatment of domestic wastewater after ozonation”, launched in 2014, compared different post-ozonation treatment technologies [2]. Similar approaches are currently being applied to investigate wastewater from industrial facilities.

#### Monitoring water pollution from non-point sources

A great number of micropollutants enter surface waters via stormwater runoff from agricultural and urban areas or via spray drift and atmospheric input. Switzerland’s Federal Office for the Environment (FOEN) and the cantonal authorities have established the National Surface Water Quality Monitoring Program (NAWA^4^) to assess and evaluate the water quality of surface waters. The Ecotox Centre contributed to special surveys (NAWA SPEZ) conducted in 2015 and 2017, which showed that small Swiss streams in agricultural areas contained numerous pesticides at concentrations exceeding legal limits. Bioassays were used to measure toxicity (potential) in ambient water samples, and results were compared to mixture risk estimated based on chemical-analytical data and water quality criteria [27, 31–33, 36].

#### Providing guidance for sediment quality monitoring

Sediments can adsorb persistent pollutants, such as heavy metals, polycyclic aromatic hydrocarbons and polychlorinated biphenyls, thereby acting as a long-term reservoir of pollution for surface waters. In Switzerland, so far no harmonised methods exist for sediment quality assessment. Therefore, the Ecotox Centre together with the FOEN has been developing a “Sediment Module” within the “Swiss Modular Stepwise Procedure (Modul-Stufen-Konzept^5^)”, which aims at establishing guidelines for ecological assessment of surface waters. The goal is to establish a comprehensive methodology for the ecotoxicological evaluation of sediment quality, which will include sampling, the development of sediment quality standards for priority chemicals, as well as—at a later stage—the application of bioassays and community indices [7, 8, 15].

#### Advancing the ecotoxicological assessment of soil

Numerous human activities lead to chemical contamination of soil, primarily via atmospheric deposition and infiltration of stormwater. In Switzerland, practically all soil contains elevated concentrations of contaminants (FOEN [16]). These can be transported into groundwater or taken up by crops and other plants. Organism communities living in soil can be directly affected and at risk, which negatively affects diversity and soil fertility. This, in turn, will reduce important ecological functions of soils such as nutrient supply, water storage and purification of water.

In the area of soil ecotoxicology, the Ecotox Centre’s overall aim is to develop concepts and recommendations for soil quality assessment. Among bioassays, the bait lamina test has proven to be a promising test [4], applicable both in the laboratory and field. Several projects have focused on testing and validating this test method in e.g. agricultural areas and shooting ranges [4, 6]. Because the interpretation of the biological response is influenced by different environmental factors, a study is currently underway to quantify the influence of soil humidity on feeding activity of relevant soil organisms. Two laboratory bioassays, a reproduction test with collemboles, and a behavioral test with earthworms, were applied in the project “Ecotoxicity of biocide-containing facade renders” (Vermeirssen et al. submitted), and for testing the effects of wood preservatives on soil organisms [5]. The Ecotox Centre has also contributed to the development of an ecotoxicity testing method using soil protists [1].

#### Making ecotoxicological methods applicable for regulators

Standardized guidelines are essential for applying bioassays in a regulatory or commercial context. A number of standardized bioassays exist and have been applied for decades, primarily for substance registration purposes or water and sediment monitoring (mostly in the USA and Canada). Numerous in vitro assays, especially those for the detection of specific mechanisms of toxicity (e.g. estrogen receptor binding, mutagenicity) are, however, not yet standardized and approved as guidelines. The Ecotox Centre is actively involved in activities for the standardization of selected aquatic bioassays according to International Standardisation Organisation (ISO) and Deutsche Industrienorm (DIN). This involves laboratory experiments for their optimization, participation in international ring tests (e.g. [13] and in expert groups. To date, three ISO standards for measuring estrogenicity were developed (ISO 19040-1 to 3; [18]; a standard for the modified algae growth assay is being produced, and an initiative was launched towards a new standard for an algae assay to measure photosystem II inhibitors [12]. As far as OECD activities are concerned, the Ecotox Centre participates in working groups on non-animal testing for endocrine disruption, and provides comments on in vitro test guidelines and validation studies.

Moving forward in new areas of ecotoxicological research:

i. The ecotoxicity of building materials is an emerging area of research. The Centre recently contributed to a report of the European Committee for Standardization (CEN) on the application of ecotoxicity tests to leachates from construction products [9]. Together with Hochschule für Technik Rapperswil, Institut für Umwelt und Verfahrenstechnik, the Ecotox Centre investigated the ecotoxicity of steel construction coatings used to prevent corrosion [37] and stormwater runoff of biocide-containing facade renders (Vermeirssen et al. submitted). The findings can contribute to the production and use of more environmentally friendly building materials.
ii. Transcription of biomarker genes in resident aquatic organisms: Modern molecular techniques offer new opportunities for monitoring pollutant impacts in exposed organisms. In 2013, the Ecotox initiated projects aimed at developing molecular tools to measure contaminant effects of wastewater effluent in resident brown trout. Cellular expression of a set of carefully selected biomarker genes was indicative of exposure to treated wastewater [14]. The Centre continues to work with research groups on advancing current approaches for evaluating the effects of pollution on resident species in aquatic and terrestrial ecosystems.
iii. DNA barcoding for community analyses: The evaluation of the quality of aquatic ecosystems based on community indices requires highly specialized expertise, is costly and time consuming. DNA barcoding for species identification promises to be a valuable approach for high-throughput biomonitoring. The Ecotox Centre is engaged in the highly collaborative Franco-Swiss Interreg Europe^6^ project SYNAQUA aimed at developing the molecular tools needed for biomonitoring diatom and oligochaete communities [39].
iv. Assessing ecotoxicological effects on benthic communities: Today, there is still a lack of adequate strategies and methods to better evaluate the ecotoxicological impact of sediment-associated chemical substances, particularly at the level of benthic communities. Assessing ecotoxicological effects on communities provides an opportunity for integrating specific and functional biodiversity in such assessments. To do this successfully, it is important to strengthen the existing methodological framework for sediment quality assessment and develop new tools and approaches to better take into account benthic communities. In 2017, the Ecotox Centre co-organized a workshop as a forum for discussion and information exchange between scientists, regulators and other stakeholders [34].

### Retrospective environmental risk assessment

Environmental hazard and risk assessment are important regulatory tools to identify and quantify risk due to chemicals, and provide the scientific basis for risk management measures.

Effect-based water quality criteria are threshold values derived for assessing the risk of chemicals based on the best available scientific information. They are necessary for assessing the risk of (known) mixtures of chemicals. The Ecotox Centre has been a major partner of the FOEN in prioritizing compounds and deriving effect-based water quality criteria following EU guidelines. So far, the Ecotox Centre has derived water quality criteria for 87 organic micropollutants and 55 of these are to be implemented in the revised Swiss Water Protection Ordinance in 2018.

Methods for modeling and evaluating predicted concentrations of chemicals present in wastewater treatment effluent were developed and applied in Switzerland and abroad [19, 21, 29].

One of the focus areas at the Ecotox Centre is the risk assessment of chemical mixtures. In collaboration with Dow Chemical Company (USA), Chris Watts Associates und WCA Environment Ltd. (both UK), Dow Europe GmbH, and the University of Lausanne, the Ecotox Centre developed advanced concepts and methods for assessing mixture toxicity in surface waters [10, 17]. These approaches were applied and validated in several case studies. The methods developed here were readily adopted by several Swiss cantonal authorities.

The Ecotox Centre also participated and actively contributed to international working groups focused on hazard and risk assessment of chemicals. Such efforts led to its involvement in a European Commission report on “Effect-based tools for water quality monitoring” [40] and the EU-wide “Estrogen Monitoring Project” [23, 26]; another led to the development of a new evaluation system for reliability and relevance assessments of study reports and peer-reviewed literature for hazard and risk assessment (Criteria for Reporting and Evaluating ecotoxicity Data, CRED; [22, 30]. The method is designed to increase the consistency of hazard and risk assessments, and—ultimately—lead to an improved quality of scientific publications. It is being readily accepted and implemented by international regulatory bodies. A similar method for improving data evaluation used for deriving sediment quality standards is currently under development at the Ecotox Centre.

### Teaching and training

Under its mandate, the Ecotox Centre organizes further education courses—mostly two 2-day courses per year. Topics range from “Introduction to Ecotoxicology” to “Science for the Regulation of Nanomaterials” and “Multiple Stressors—Effects of Chemicals and Environmental Factors on Organisms and Ecosystems”. Recently, 1-day training courses were established to transfer specific skills (e.g. deriving environmental quality standards) to environmental professionals, and the Centre regularly contributes to advanced education programmes at universities, especially universities of applied sciences. In this, it collaborates frequently with its “sister institution”, the Swiss Centre for Applied Human Toxicology (SCAHT^7^). The courses are advertised directly by the Ecotox Centre, as well as through Eawag’s PEAK programme and the further education programme at the University of Lausanne. Depending on the topic, the majority of course participants come from private industry, cantonal agencies, federal agencies and academia. The remaining contingent consists of members of professional and non-profit (primarily environmental) organizations and private persons.

The Ecotox Centre participates in the Eawag apprenticeship program for laboratory technicians, and offers internships and bachelor/master thesis projects to students. To date over 40 university students have realized internships, or performed their bachelor/master thesis research at the Centre.

### Informing, publishing and reaching out

Communication of research results to experts and basic concepts of ecotoxicology to the public are important aspects of the Ecotox Centre’s mission. Since its foundation, the Centre has published over 104 articles in peer-reviewed and 29 articles in non-peer-reviewed journals, primarily *Aqua & Gas.*^8^ Centre scientists have contributed a total of 101 posters and 94 platform presentations to scientific conferences. As a service to environmental professionals and the interested public, fact sheets are produced based on current interest in specific topics and made available on the Ecotox Centre website in German, French and English.^9^ They are intended for providing an informative overview on specific topics.

The Ecotox Centre participates in events for students, and regularly hosts several visits by school classes or technical universities per year. Centre scientists also give presentations upon request.

**Fig. 3:**
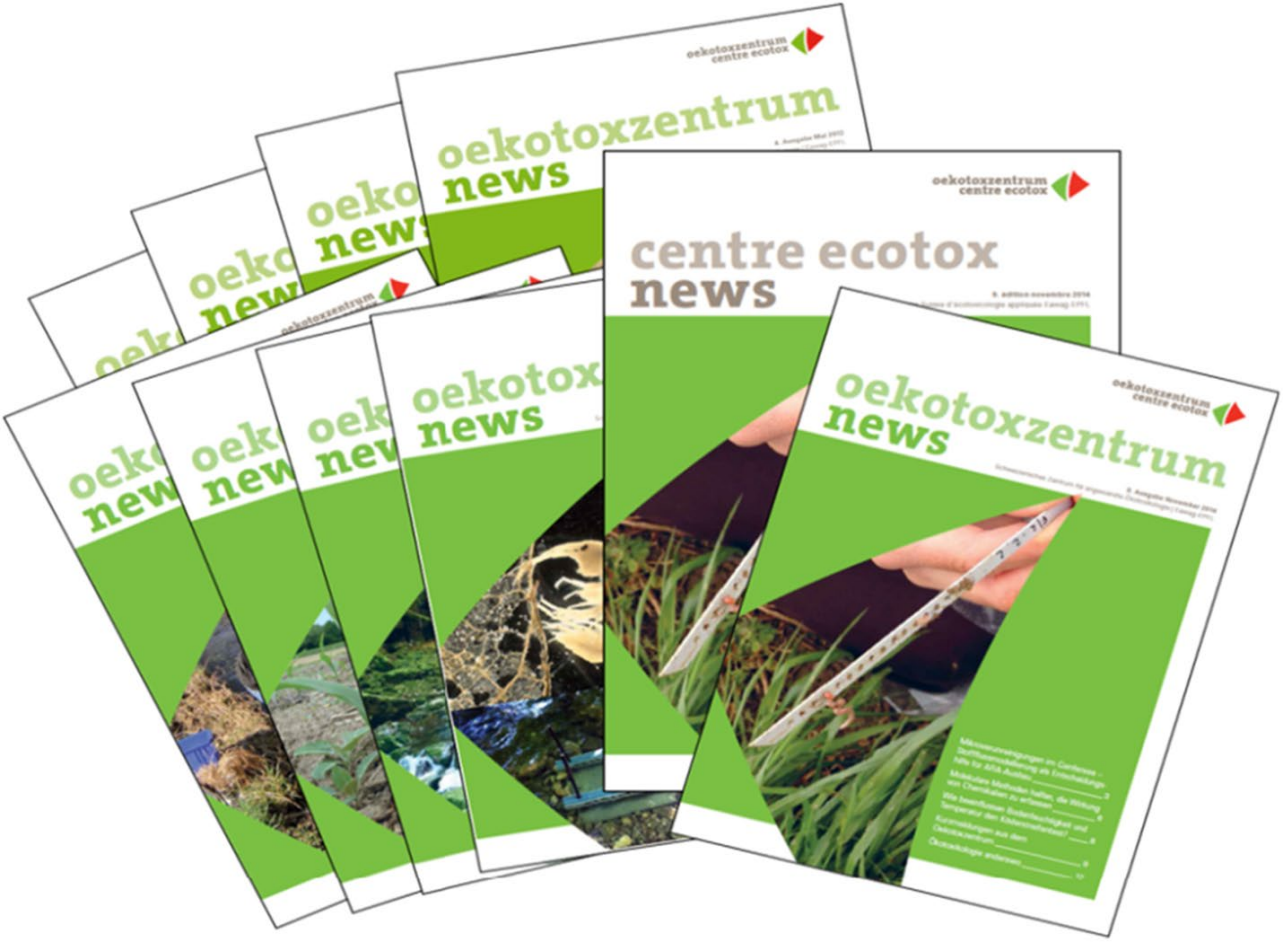
The Oekotoxzentrum/Centre Ecotox News, the Ecotox Centre’s newsletter, appears twice a year

Since 2010, the Centre’s bilingual (German, French) newsletter, the Oekotoxzentrum/Centre Ecotox News^10^ has been published twice a year (Fig. 3). It informs about projects performed at the Ecotox Centre, new developments, events and courses, and provides information on important news and publications in the field. The News are available free of charge as pdf on the website or can be obtained in printed form by mail. Currently, the Ecotox Centre News are sent to 890 subscribers.

### Consulting and contributing to expert groups

Each year, the Ecotox Centre receives about 250 requests for information from stakeholder groups, the public and media; topics range from questions concerning water quality criteria to the effects of micropollutants, especially endocrine disruptors, pharmaceuticals and pesticides.

Currently, Centre scientists actively participate in approximately 40 national and international expert groups, such as—to name a few—*Leitungsgruppe Gewässerbeurteilung Schweiz,* the *European Sediment Network (SedNet)* steering group, science advisory boards of *Commission international pour la protection des eaux du Léman* (CIPEL) and *Schweizerische Gesellschaft für Hydrologie und Limnologie*, the *OECD Test Guideline Programme, OECD Endocrine Disrupter Testing and Assessment Advisory Group* and the *Expert Advisory Group on Endocrine Disruptors* of the EU Commission. Other important activities include the Centre’s involvement in NORMAN, a network of reference laboratories, research centers and related organisations for monitoring of emerging environmental substances, where it participates in working groups on the prioritization of chemicals, the use of bioassays and biomarkers in, and the application of passive samplers to water quality monitoring.

## Concluding remarks

Since its foundation in October 2008, the Ecotox Centre has carried out numerous projects whose results directly informed regulators and practitioners. It organized 18 further education courses, published 41 reports and over 104 peer-reviewed papers, and responded to thousands of questions on a variety of ecotoxicological topics. Centre scientists have supervised over 40 students in their research projects and internships, and contributed their expertise to the curriculum of several universities in Switzerland. To be able to do this, availability of continuous and reliable funding has been utterly important. It has become quite obvious that the Swiss Parliament’s decision to fund the Ecotox Centre was wise, future oriented and forward looking. As society increasingly recognizes that toxic effects of chemicals in the environment can have far-reaching consequences for ecosystems and human health, the demand for ecotoxicological expertise is expected to increase. It would, therefore, be prudent to establish more university programs and provide courses in this field, to produce the contingent of experts in ecotoxicology needed to meet the challenges of the future. It is my hope that other countries may follow Switzerland’s example in establishing such a government funded Centre for bridging the gap between academic research and practical application in the field of ecotoxicology.

## Footnotes

1 http://www.ecotoxcentre.ch.

2 Bericht des Bundesrates über die unabhängige Toxikologie-Forschung in der Schweiz, 2.5.2007, S. 3772 http://www.admin.ch/ch/d/ff/2007/3747.pdf.

3 Der Schweizer Bundesrat (2014). Gewässerschutzverordnung (GSchV) 814.201. https://www.admin.ch/opc/en/classified-compilation/19983281/index.html.

4 https://www.bafu.admin.ch/bafu/en/home/topics/water/state/water–monitoring-networks/national-surface-water-quality-monitoring-programme–nawa-html.

5 http://www.modul-stufen-konzept.ch/index_EN.

6 http://www.interregeurope.eu/.

7 http://www.scaht.org/.

8 www.aquaetgas.ch/.

9 http://www.ecotoxcentre.ch/news-publications/info-sheets/.

10 http://www.oekotoxzentrum.ch/news-publikationen/oekotoxzentrum-news/.
